# Aloin Suppresses Lipopolysaccharide-Induced Inflammatory Response and Apoptosis by Inhibiting the Activation of NF-κB

**DOI:** 10.3390/molecules23030517

**Published:** 2018-02-26

**Authors:** Xuan Luo, Haowei Zhang, Xiduan Wei, Mengjuan Shi, Ping Fan, Weidong Xie, Yaou Zhang, Naihan Xu

**Affiliations:** 1 Key Lab in Healthy Science and Technology, Division of Life Science, Graduate School at Shenzhen, Tsinghua University, Shenzhen 518055, China; lxuan0721@163.com (X.L.); weixiduan@126.com (X.W.); 18700809087@163.com (M.S.); pingf01@126.com (P.F.); xiewd@tsinghua.edu.cn (W.X.); zhangyo@sz.tsinghua.edu.cn (Y.Z.); 2 Open FIESTA Center, Tsinghua University, Shenzhen 518055, China; zhwjack@hotmail.com

**Keywords:** inflammation, aloin, NF-κB, macrophages, apoptosis

## Abstract

Numerous herbal-derived natural products are excellent anti-inflammatory agents. Several studies have reported that aloin, the major anthraquinone glycoside obtained from the *Aloe* species, exhibits anti-inflammatory activity. However, the molecular mechanism of this activity is not well understood. In this report, we found that aloin suppresses lipopolysaccharide-induced pro-inflammatory cytokine secretion and nitric oxide production, and downregulates the expression of tumor necrosis factor alpha (TNF-α), interleukin 6 (IL-6), inducible nitric oxide synthase (iNOS), and cyclooxygenase-2 (COX-2). Aloin inhibits the phosphorylation and acetylation of the NF-κB p65 subunit by suppressing the upstream kinases p38 and Msk1, preventing LPS-induced p65 translocation to the nucleus. We have also shown that aloin inhibits LPS-induced caspase-3 activation and apoptotic cell death. Collectively, these findings suggest that aloin effectively suppresses the inflammatory response, primarily through the inhibition of NF-κB signaling.

## 1. Introduction

In response to infection or tissue injury, immune cells initiate signaling cascades that trigger the release of various pro-inflammatory cytokines to combat tissue injury or pathogen invasion. Macrophages play important roles in the initiation, maintenance, and resolution of inflammation. Macrophage dysfunction can cause a variety of human inflammatory disorders, such as microbial infection, sepsis-related multiple organ failure, tumorigenesis, cardiovascular and metabolic diseases, and autoimmune diseases. 

The transcription factor NF-κB plays a vital role in inflammatory and immune responses. NF-κB is highly activated at sites of inflammation in many diseases, and can induce the transcription of pro-inflammatory cytokines and chemokines, as well as various target genes, including inducible nitric oxide synthase (iNOS) and cyclooxygenase-2 (COX-2) [1,2,3]. The feasibility and efficacy of specific inhibition of NF-kB activity has been shown in several animal models of inflammatory disease [4,5,6]. The NF-κB complex contains sites for post-translational modifications, which are important for activation and crosstalk with other signaling pathways [1,7,8]. The phosphorylation and acetylation of the p65/RelA subunit are crucial post-translational modifications that are required for NF-κB activation. The phosphorylation of p65 by PKAc, MSK1, RSK1, IKKα/β, and PKCζ enhances its interaction with the co-activator p300/CBP, and stimulates the activity of NF-κB [9,10]. The p65 subunit of NF-κB is also known to be activated in an acetylation-dependent manner in response to inflammatory stimuli. The site-specific acetylation of p65 differentially regulates the distinct biological activities of the NF-κB transcription factor complex [11]. For instance, the acetylation of lysine 310 is indispensable for the full transcriptional activity of p65. However, lysine 122 and 123 acetylation regulate the IκBα-mediated attenuation of NF-κB transcriptional activity, which is an important process that restores the latent state in post-induced cells [11,12,13].

Natural products are important sources of drugs against a variety of diseases, such as cancer and infectious diseases. Many natural products that have in anti-cancer and anti-inflammatory activity have been shown to inhibit NF-κB. Aloin, which is also known as barbaloin, is the major anthraquinone glycoside obtained from the *Aloe* species. Several lines of evidence demonstrate that aloin exhibits anti-oxidative and anti-inflammatory activities in rats and murine macrophages [14,15,16,17,18]. Aloin also shows a pronounced anti-proliferative effect, the treatment of aloin-induced cell cycle arrest, and apoptotic cell death in several human cancer cell lines [19,20,21,22]. Moreover, a cell line-derived xenograft mouse model indicates that aloin inhibits tumor angiogenesis and growth via blocking STAT3 activation [23]. In this study, we evaluate the potential anti-inflammatory activity of aloin in RAW264.7 cells. We show that aloin protects macrophages from a lipopolysaccharide (LPS)-induced inflammatory response by inhibiting the NF-κB signaling pathway. Aloin blocks the phosphorylation, acetylation, and nuclear translocation of the NF-κB p65 subunit. Aloin also downregulates stress-related gene expression, and protects macrophages from an LPS-induced inflammatory response and apoptotic cell death.

## 2. Results

### 2.1. Effects of Aloin on Cell Viability in Macrophages

**Figure 1:**
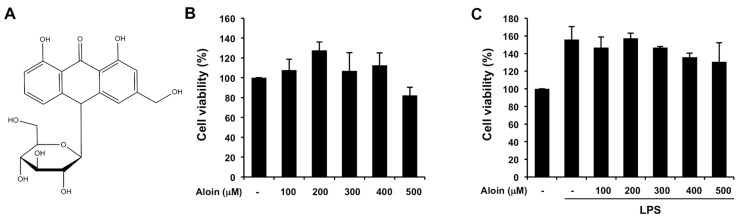
Aloin does not affect cell growth in murine macrophages: (**A**) Chemical structure of aloin; (**B**) RAW 264.7 cells were treated with various concentrations of aloin (100 μM, 200 μM, 300 μM, 400 μM, and 500 μM) for 24 h. The total number of viable cells was determined by methyl thiazolyl tetrazolium (MTT) assay. Values are mean ± SD of three independent experiments; (**C**) RAW 264.7 cells were treated with various concentrations of aloin and lipopolysaccharide (LPS) (100 ng/mL) for 24 h. The total number of viable cells was calculated. Values ARE mean ± SD of three independent experiments.

To evaluate the cytotoxicity of aloin, RAW264.7 cells were incubated with different concentrations of aloin for 24 h, in the presence or absence of lipopolysaccharide (LPS), after which cell viability was determined by methyl thiazolyl tetrazolium (MTT) assay. High concentrations of aloin (up to 500 μM) did not show significant cytotoxicity in RAW264.7 cells (Figure 1A,B). Rather, the addition of LPS enhanced cell proliferation in macrophages, and aloin had no effect on LPS-induced cell proliferation when compared with the control cells (Figure 1C). 

### 2.2. Aloin Inhibits LPS-Induced IL-6 and TNF-α Expression

**Figure 2:**
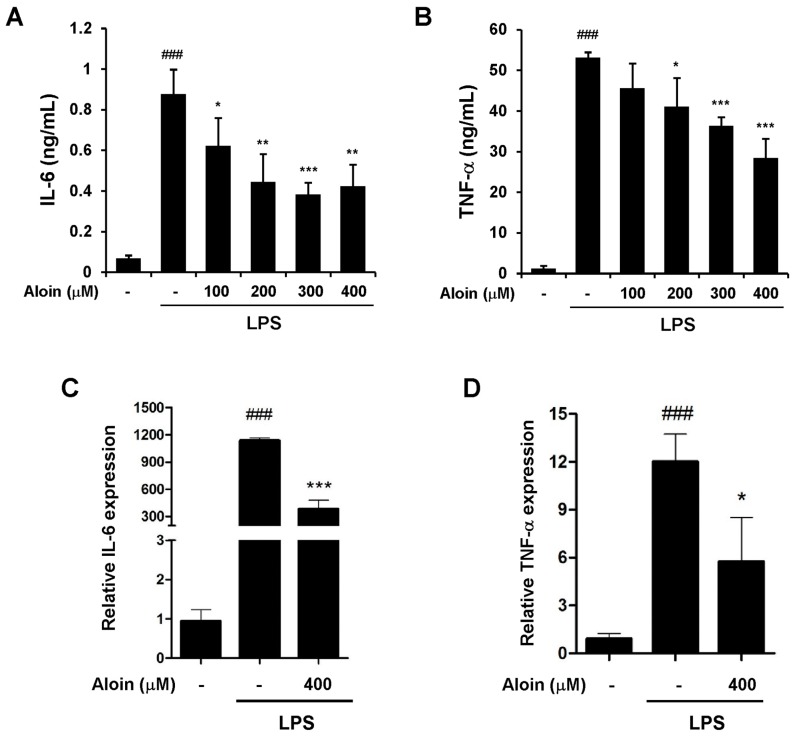
Aloin inhibits the LPS-induced expression of IL-6 and TNF-α RAW 264.7 cells were pre-treated with aloin for 2 h prior to LPS (100 ng/mL) stimulation. Cell-free supernatants were collected to detect (**A**) Interleukin 6 (IL-6) and (**B**) Tumor necrosis factor alpha (TNF-α) concentrations via ELISA after LPS treatment for 9 h and 24 h, respectively. The mRNA expression levels of (**C**) IL-6 and (**D**) TNF-α were determined by qPCR in RAW264.7 cells pre-treated with aloin (400 μM) followed by LPS (100 ng/mL) stimulation for 6 h. The data shown are the means ± SD of three experiments. ^###^ *p* < 0.001 is significantly different from the control. * *p* < 0.05, ** *p* < 0.01, and *** *p* < 0.001 are different from the LPS alone.

To investigate the effect of aloin on the LPS-induced inflammatory response, we measured the secretion of inflammatory cytokines in RAW 264.7 cells. LPS dramatically stimulated the production of pro-inflammatory cytokines, such as tumor necrosis factor alpha (TNF-α) and interleukin 6 (IL-6), as compared with control cells. Aloin efficiently suppressed the elevated cytokine levels in LPS-treated macrophages in a dose-dependent manner (Figure 2A,B). We also investigated whether or not aloin could inhibit the LPS-induced transcription of TNF-α and IL-6 by real-time PCR. Total RNA was extracted six hours after LPS and/or aloin treatment, and aloin was found to markedly reduce LPS-induced TNF-α and IL-6 transcription (Figure 2C,D). These data indicate that aloin inhibits the LPS-induced inflammatory response in murine macrophages.

### 2.3. Aloin Attenuates LPS-Induced NO Production

**Figure 3:**
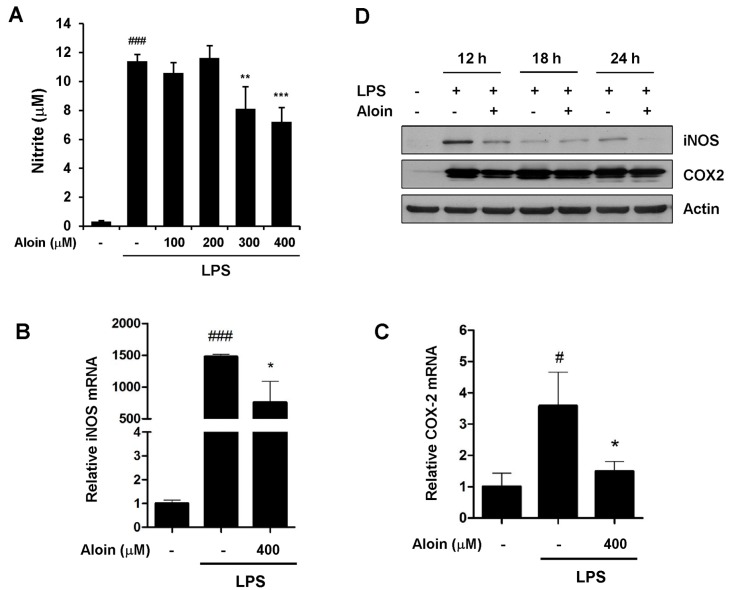
Aloin inhibits nitric oxide (NO) production and the expression of cyclooxygenase-2 (COX-2) and inducible nitric oxide synthase (iNOS). (**A**) RAW 264.7 cells were pre-treated with aloin for 2 h prior to LPS (100 ng/mL) treatment. Cell-free supernatants were collected to determine NO production using the Griess reaction after LPS treatment for 24 h. The mRNA levels of (**B**) iNOS and (**C**) COX2 were determined by qPCR; (**D**) RAW 264.7 cells were pre-treated with or without 400 μM aloin for 2 h, and then stimulated with LPS (100 ng/mL) for 12 h, 18 h and 24 h. Levels of iNOS and COX-2 proteins were determined by Western blotting. The results shown are the means ± SD of three experiments. ^#^*p* < 0.05 and ^###^ *p* < 0.001 are significantly different from the control. * *p* < 0.05, ** *p* < 0.01, and *** *p* < 0.001 are different from the LPS alone.

Because nitric oxide (NO) is recognized as a mediator and regulator of inflammatory responses, we next determined the effect of aloin on NO production. As shown in Figure 3A, NO levels were dramatically increased from resting up to ~10 μM following LPS treatment. The dose-dependent inhibition of NO production was observed when RAW264.7 cells were pre-treated with different concentrations of aloin (Figure 3A). 

### 2.4. Aloin Suppresses iNOS and COX-2 Expression

The inducible nitric oxide synthase (iNOS) gene is involved in the immune response and NO production. Cyclooxygenase-2 (COX-2) is rapidly induced by a variety of extracellular and intracellular stimuli, and plays a key role in promoting inflammation. We determined the effect of aloin on the expression of iNOS and COX-2 by quantitative RT-PCR and immunoblot analyses. The mRNA levels of both iNOS and COX-2 were markedly upregulated following LPS treatment (Figure 3B,C). Notably, aloin reduced LPS-induced iNOS protein expression, while it had little effect on COX-2 protein levels (Figure 3D). These results indicate that aloin suppresses LPS-induced NO production, primarily due to the aloin-mediated downregulation of iNOS. 

### 2.5. Aloin Inhibits LPS-Induced p65 Phosphorylation and Acetylation

**Figure 4:**
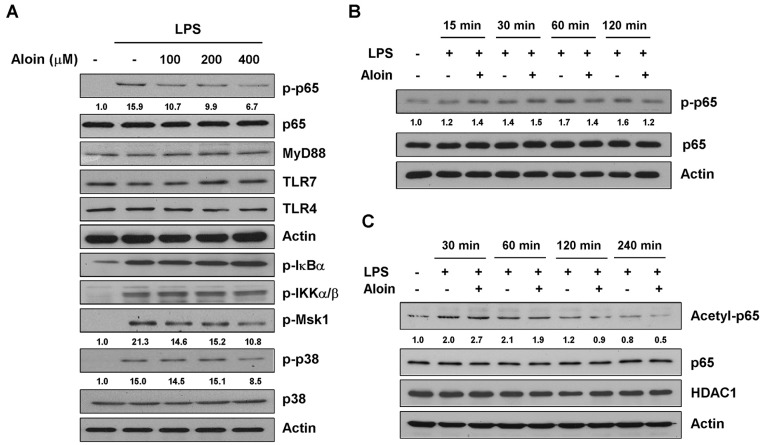
Aloin inhibits LPS-induced NF-κB p65 phosphorylation and acetylation. (**A**) RAW 264.7 cells were pre-treated with various concentrations of aloin for 2 h, and stimulated with LPS (100 ng/mL) for 2 h. The protein expression levels of phospho-p65, total p65, MyD88, TLR4, TLR7, phosphor-IκBα, phospho-IKKα/β, phospho-Msk1, phospho-p38 and total p38 was determined by Western blotting; (**B**) Cells were pre-treated with or without aloin (400 μM) for 2 h, and stimulated with LPS (100 ng/mL) for 15 min, 30 min, 60 min, or 120 min. The protein levels of phospho-p65 and total p65 were analyzed by Western blotting; (**C**) Immunoblotting analysis of acetylated p65, total p65, and HDAC1 in RAW 264.7 cells treated with aloin and LPS at indicated time points.

The LPS-induced activation of macrophages involves the Toll-like receptors. The binding of LPS to TLR4 leads to the activation of NF-κB through the recruitment and activation of MyD88 and other adaptor proteins [24]. We examined the expression of Toll-like receptors and MyD88 in RAW264.7 cells, and found that aloin did not affect the protein levels of TLR4, TLR7, and MyD88 in LPS-stimulated macrophages (Figure 4A). 

Since NF-κB plays an important role in the regulation of a variety of key inflammatory mediators, we sought to determine whether aloin could inhibit LPS-induced NF-κB activation. Immunoblotting revealed that aloin reduced LPS-induced phosphorylation of p65 in a dose and time-dependent manner, without affecting total p65 expression. Multiple upstream signaling events can trigger p65 phosphorylation, and further enhance NF-κB transcriptional activity. To examine enzyme specificity, we investigated the upstream kinases, including IKKα/β, IκB, Msk1, and p38. Aloin suppressed the phosphorylation of both Msk1 and p38 in a dose-dependent manner, but had little effect on IKKα/β and IκB phosphorylation (Figure 4A,B), indicating that aloin inhibits p65 phosphorylation mainly through the upstream kinases p38 and Msk1.

As acetylation of p65 also functions as a molecular switch that controls the transcriptional activation of NF-κB, we next assessed the effect of aloin on LPS-induced p65 acetylation in RAW 264.7 cells. Cells were pre-treated with aloin for two hours, and stimulated with LPS at different time points. As shown in Figure 4C, aloin treatment suppressed LPS-induced acetylation of p65 in a time-dependent manner. The protein level of HDAC1 did not change during treatment. These results suggest that aloin prevents the phosphorylation and acetylation of p65 during an inflammatory response. 

### 2.6. Aloin Inhibits LPS-Induced Nuclear Translocation of p65

**Figure 5:**
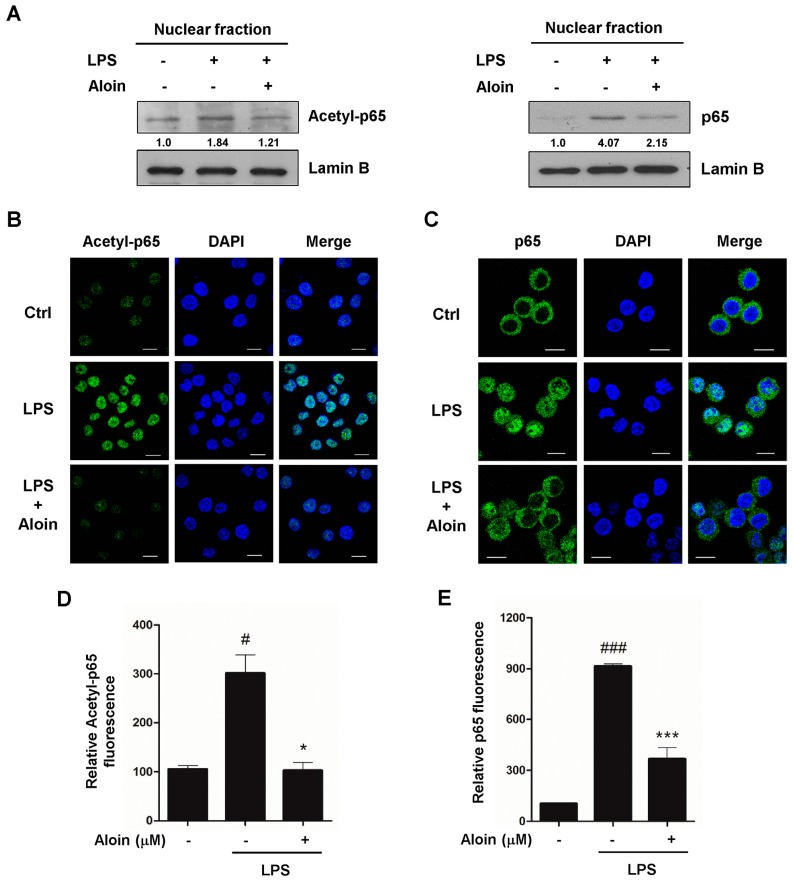
Aloin inhibits LPS-induced NF-κB p65 nuclear translocation. (**A**) RAW 264.7 cells were pre-treated with or without aloin (400 μM) for 2 h, and then stimulated with LPS (100 ng/mL) for 2 h. Protein levels of acetylated p65 and total p65 in the nuclear fractions were analyzed by immunoblotting. Lamin B was used as a marker for nuclear fraction. RAW 264.7 cells treated with or without aloin and LPS were fixed and immunostained with (**B**) acetylated p65 or (**C**) total p65 antibodies, and fluorescent images were captured by confocal microscopy. Scale bar, 10 μm. At least 200 cells were counted for each sample; (**D**) Quantification of acetyl-p65 and (**E**) total p65 fluorescent intensities in RAW 264.7 cells. Data shown are the means ± SD of three experiments. ^#^ *p* < 0.05 and ^###^ *p* < 0.001 are different from the control. * *p* < 0.05 and *** *p* < 0.001 are different from the LPS alone.

Following inflammatory stimulation, NF-κB is activated by post-translational modifications, and translocates into the nucleus to induce the transcription of pro-inflammatory genes. To determine the effect of aloin on the translocation of the NF-κB p65 subunit, RAW 264.7 cells were pre-treated with aloin, and then stimulated with LPS. Nuclear fractions were then extracted, and subsequent Western blot analysis was performed using antibodies against acetylated p65 and total p65. As shown in Figure 5A, the levels of both acetylated p65 and total p65 proteins were markedly increased in LPS-stimulated cells, and aloin treatment significantly reduced the nuclear accumulation of p65. 

Next, we performed an immunofluorescence assay to examine the nuclear localization of acetylated p65. First, p65 was acetylated at a very low level in the control cells, and LPS treatment dramatically increased nuclear staining of acetylated p65, while aloin markedly reduced the nuclear localization of acetylated p65 (Figure 5B,D). The nuclear translocation of p65 was also confirmed by immunostaining with a total p65 antibody. As expected, LPS treatment induced the translocation of p65 from the cytoplasm to the nucleus, and aloin prevented LPS-induced nuclear translocation in RAW 264.7 cells (Figure 5C,E). 

### 2.7. Aloin Prevents LPS-Induced Apoptotic Cell Death

**Figure 6:**
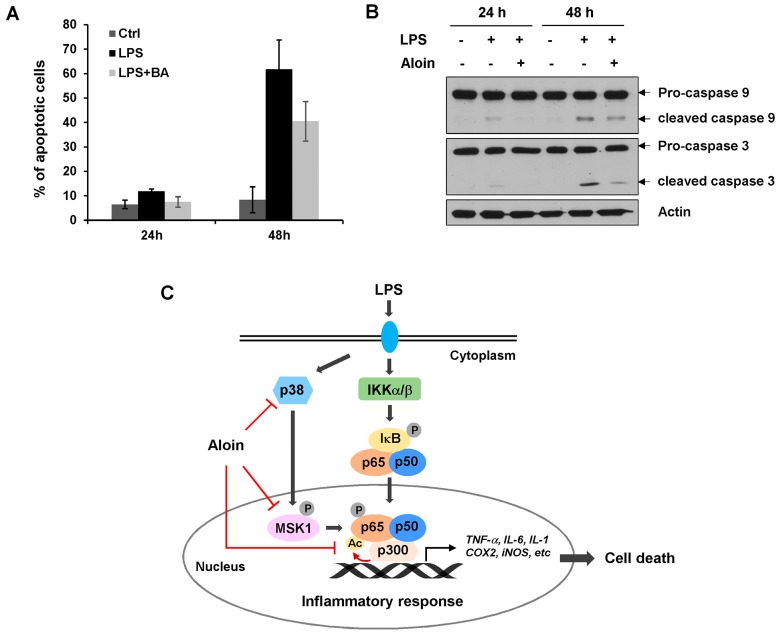
Aloin inhibits LPS-induced apoptotic cell death. (**A**) RAW 264.7 cells were pre-treated with or without aloin (400 μM) for 2 h, and then stimulated with LPS (100 ng/mL) for 24 h or 48 h. Cells were collected and stained with Annexin V/PI, the percentage of Annexin-V positive cells was analyzed by flow cytometry analysis Data shown are the means ± SD of three experiments; (**B**) The protein expression levels of pro-caspase 9, cleaved caspase-9, pro-caspase 3, and cleaved caspase 3 were determined by immunoblotting analysis. β-actin was used as a loading control; (**C**) Aloin prevented the LPS-induced inflammatory response by inhibiting NF-κB signaling. Aloin attenuated LPS-induced p65 post-translational modifications by inhibiting p38 and MSK1-mediated phosphorylation and p300-mediated acetylation. This in turn prevented p65 nuclear translocation, and downregulated NF-κB mediated gene expression, including pro-inflammatory cytokines, and genes involved in apoptosis.

It has been reported that LPS induces apoptosis in several cell types. To evaluate the effect of aloin on LPS-induced apoptosis in macrophages, we performed annexin V/PI flow cytometry analysis to measure apoptotic cell death. Annexin-V positive cells increased with the time of LPS treatment. At 48 h after LPS administration, 62% of the cell population were apoptotic. LPS-induced apoptosis was markedly reduced by aloin treatment (Figure 6A). Immunoblotting analysis revealed that LPS caused an obvious increase in the levels of cleaved caspase-9 and caspase-3, indicating that LPS induces caspase-mediated apoptosis. Supporting the flow cytometry results, the LPS-induced cleavage of caspase-9 and caspase-3 were largely prevented by aloin (Figure 6B).

## 3. Discussion

Although aloin is known to have anti-inflammatory and anti-oxidative activities, the molecular basis of the anti-inflammatory effects of aloin is still unclear. In this study, we demonstrate that aloin, a natural product extracted from the *Aloe* species, is an effective anti-inflammatory agent. Aloin inhibits the LPS-induced expression and secretion of the pro-inflammatory cytokines IL-6 and TNF-α. Aloin blocks the phosphorylation, acetylation, and nuclear translocation of the NF-κB p65 subunit. Aloin also inhibits NO production, and downregulates stress-related gene expression. These data indicate that aloin induced anti-inflammatory activity is mediated through the inhibition of NF-κB signaling (Figure 6C).

The phosphorylation and acetylation of p65 are critical to NF-κB-dependent activation. LPS can activate the IκB kinase (IKK) enzyme complex, which phosphorylates IκB proteins. The phosphorylation of IκB leads to its ubiquitination and degradation, releasing NF-κB/p65 complexes. NF-κB/p65 complexes are further activated by post-translational modifications and translocate to the nucleus [9,25,26]. We examined the upstream kinases that control p65 phosphorylation. We found that aloin inhibits the LPS-induced phosphorylation of p38 and Msk1. The p38 MAPK is crucial for LPS-induced cytokine gene expression. p38 induces the phosphorylation of histone H3, and increases DNA accessibility for NF-κB binding at specific promoters. p38 also phosphorylates and activates Msk1, which increases transcription through the phosphorylation of p65 at serine 276. p65 Ser 276 phosphorylation promotes its own interaction with the cofactors CREB/p300, which in turn acetylates p65 itself, as well as histones at NF-κB-bound promoters [27,28,29]. We also found that aloin suppressed LPS-induced p65 acetylation, and markedly reduced the nuclear translocation of p65 in LPS-stimulated macrophages. Thus, our study has uncovered a feasible mechanism in which aloin suppresses p65 post-translational modifications and nuclear translocation, and consequently attenuates the level of NF-κB-dependent transcription and the inflammatory response.

Nitric oxide plays an important role in mediating many aspects of inflammatory responses. iNOS is one of the three enzymes that catalyze the production of NO from l-arginine [30]. It is an important factor in clearing parasites and bacterial infections. The level of NO produced by iNOS is predominantly regulated at the transcriptional level. LPS, cytokines, and oxidative stress have been shown to induce iNOS expression through the activation of NF-κB [31,32]. COX-2 plays a key role in the inflammatory response. A variety of extracellular and intracellular stimuli, including LPS, rapidly induce COX-2 expression [33]. NF-κB has been shown to control the induction of COX-2 transcription [34,35]. Here, we demonstrated that aloin markedly suppressed the transcription of iNOS and COX2. Aloin also decreased the level of LPS-induced NO production in RAW 264.7 cells in a dose-dependent manner. Since NF-κB plays a pivotal role in regulating the expression of *iNOS and COX-2*, we conclude that aloin inhibits LPS-induced inflammatory gene expression by suppressing NF-κB transcriptional activity. 

Several studies have shown that LPS induces apoptosis in different cell types, including macrophages [36,37,38,39]. Apoptosis has been involved in multi-organ failure during septic shock [40,41]. Multiple molecular mechanisms are involved in LPS-induced apoptosis. The deleterious effects of LPS are associated with the secretion of TNF-α and NO production. Exposure to endogenous or exogenous NO, or treatment with TNF-α, are sufficient to induce apoptosis [39,42,43]. LPS induces macrophage apoptosis via two pathways; one is through the mitochondrial apoptotic cascade leading to caspase-3 activation, while the other is mediated by death receptor and caspase-8 activation [36,44,45]. Since aloin efficiently inhibits the LPS-induced inflammatory response, we investigated whether aloin played a protective effect on LPS-induced apoptosis. Both flow cytometry and immunoblotting revealed that aloin remarkably reduced the number of Annexin-V positive cells and caspase-3 cleavage, indicating that aloin has anti-apoptotic activity against LPS-induced cell death in murine macrophages. Collectively, the results of this study suggest that aloin is an effective anti-inflammatory and anti-apoptotic agent, and acts primarily through the inhibition of the NF-κB signaling pathway. 

## 4. Materials and Methods

### 4.1. Cell Culture and Reagents

RAW 264.7 cells (murine macrophage cell line) were purchased from the Cell Library of Chinese Academy of Sciences. Cells were cultured in Dulbecco modified Eagle medium (Invitrogen, Thermofisher Scientific, Waltham, MA, USA), and supplemented with 10% fetal bovine serum (Thermofisher Scientific, Waltham, MA, USA) in a 5% CO_2_-humidified incubator at 37 °C.

Aloin (≥97%) was purchased from Shanghai Aladdin Biochemical Technology Co., Ltd. (Shanghai, China). Dimethyl sulfoxide (DMSO), lipopolysaccharide (LPS, from *Escherichia coli*) and methyl thiazolyl tetrazolium (MTT) were purchased from Sigma Chemical Co. (St. Louis, MO, USA). Aloin was dissolved in DMSO. Mouse TNF-α enzyme-linked immunosorbent assay (ELISA) and IL-6 ELISA kits were purchased from Dakewe Biotech Co., Ltd. (Shenzhen, China). The Griess reagent was purchased from the Beyotime Institute of Biotechnology (Shanghai, China). The nucleoprotein extraction kit was obtained from Sangon Biotechnology (Shanghai, China). The antibodies against iNOS, COX-2, phosphor-IκB-α (Ser32), phosphor-IKKα/β (Ser176/180), phosphor-GSK-3β (Ser9), phosphor-Msk1 (Thr581), p38, phosphor-p38 MAPK (Thr180/Tyr182), NF-κB p65, phosphor-NF-κB p65 (Ser536), Acetyl-p65 (Lys310), HDAC1, and β-actin were purchased from Cell Signaling Technology (Danvers, MA, USA). 

### 4.2. Cell Viability Assay

Cell viability was evaluated using a MTT assay. RAW 264.7 cells were seeded into 96-well plates at a density of 7 × 10^3^ cells per well, and maintained at 37 °C for 24 h. The cells were exposed to various concentrations of aloin (100 μM, 200 μM, 300 μM, 400 μM, and 500 μM) and stimulated with LPS (100 ng/mL) for 24 h. After 24 h of incubation, the MTT (5 mg/mL) solution was added to each well, and incubated for another 4 h. Then, 100 μL of DMSO was added to each well to dissolve the formazan crystals that formed, and the absorbance of the solution was measured at 490 nm. Cell viability was determined from the absorbance value, and compared with that of the untreated control group. All of the experiments were performed in triplicate.

### 4.3. Quantitative Real-Time PCR

Cells were seeded in six-well plates, treated with or without aloin for 2 h, and then stimulated with LPS (100 ng/mL) for 24 h. Total RNA was extracted and reverse transcribed, as previously described [46]. The expression of *iNOS*, *COX-2*, *TNF-α*, and *IL-6* mRNA was normalized to β-actin. The following primers were used for PCR analysis: iNOS forward 5′-GTTCTCAGCCCAACAATACAAGA-3′, iNOS reverse 5′-GTGGACGGGTCGATGTCAC-3′; COX-2 forward 5′-CCCCATTAGCAGCCAGTT-3′, COX-2 reverse 5′-CATTCCCCACGGTTTTGA-3′; TNF-α forward 5′-GGAACACGTCGTGGGATAATG-3′, TNF-α reverse 5′-GGCAGACTTTGGATGCTTCTT-3′; IL-6 forward 5′-GGCGGATCGGATGTTGTGAT-3′, IL-6 reverse 5′-GGACCCCAGACAATCGGTTG-3′; β-actin forward 5′-TCGTGCGTGACATCAAAGA-3′, β-actin reverse 5′-CATACCCAAGAAGGAAGGCT-3′.

### 4.4. Nitric Oxide Measurement

Cells were plated in 24-well plates and pre-treated with different concentrations of aloin (100 μM, 200 μM, 300 μM, and 400 μM) for 2 h, followed by treatment with LPS (100 ng/mL) for another 24 h. Cell-free supernatants were collected for the determination of NO content using a commercially available kit based on the Griess reaction (Beyotime).

### 4.5. Cytokine Measurement

Cells were plated in 24-well plates, and pre-treated with different concentrations of aloin (100 μM, 200 μM, 300 μM, and 400 μM) for 2 h, then incubated with LPS (100 ng/mL) for different time periods. Cell-free supernatants were collected for the determination of TNF-α and IL-6 concentrations via ELISA analysis (Dakewe), according the manufacturer’s protocols. 

### 4.6. Western Blot Analysis

Cells were lysed in an ice-cold whole cell extract buffer (50mM Tris-HCl, pH 8.0, 4 M urea and 1% Triton X-100) supplemented with a protease inhibitor mixture (Roche). Cell extracts were resolved by SDS-PAGE, and transferred to a nitrocellulose membrane. Membranes were probed with primary antibodies against iNOS, COX-2, p-IκB-α, p-IKKα/β, p-GSK3β, p-Msk1, p38, p-p38, p65, p-p65, acetyl-p65, HDAC1, and β-actin, followed by incubation with horseradish peroxidase (HRP)-conjugated secondary antibodies (KPL). Protein bands were developed using enhanced chemiluminescence (ECL) Western Blotting Substrate (Thermo Scientific, Waltham, MA, USA).

### 4.7. Immunofluorescence

RAW 264.7 cells were seeded in 12-well plates, pretreated with aloin (400 μM) for 2 h, and then stimulated with LPS (100 ng/mL) for another 2 h. Cells were fixed in freshly prepared 4% paraformaldehyde solution for 15 min, washed three times with phosphate-buffered saline (PBS), and permeabilized with 0.25% Triton X-100 for 10 min. The fixed cell preparations were blocked in 3% BSA for 1 h, and incubated with p65 (Cell Signaling Technology) or acetyl-p65 (GeneTex, Irvine, CA, USA) antibodies for 1 h at room temperature. The stained cells were washed and incubated with Alexa Fluor 488 conjugated secondary antibody (Invitrogen) for 1 h. Fluorescent images were acquired using an Olympus FV1000 confocal microscope (Tokyo, Japan).

### 4.8. Flow Cytometry

RAW 264.7 cells were placed in six-well plates, and treated with or without aloin (400 μM) for 2 h prior to LPS (100 ng/mL) treatment for 24 h and 48 h. Cells were harvested and processed using the Dead Cell Apoptosis Kit with Annexin V Alexa Fluor™ 488 and propidium iodide (Invitrogen). Cells were counted by using a BD Accuri C6 Flow cytometer. 

### 4.9. Statistical Analyses

Data are expressed as mean ± SD of three independent experiments. Statistical analyses were performed using two-tailed student’s *t*-test. Differences were considered significant if *p* < 0.05.
